# Long-Term Effect of Salt Substitute on All-Cause and Cardiovascular Disease Mortality: An Exploratory Follow-Up of a Randomized Controlled Trial

**DOI:** 10.3389/fcvm.2021.645902

**Published:** 2021-05-17

**Authors:** Hao Sun, Bing Ma, Xiaomei Wu, Hailong Wang, Bo Zhou

**Affiliations:** ^1^Department of Clinical Epidemiology and Evidence-Based Medicine, The First Affiliated Hospital, China Medical University, Shenyang, China; ^2^Institute of Cardiovascular Diseases, The First Hospital of China Medical University, Shenyang, China

**Keywords:** salt substitute, all-cause mortality, hypertension, stroke mortality, CVD mortality

## Abstract

**Background:** Salt substitute, a strategy for salt reduction, has been shown to decrease blood pressure and the incidence of hypertension. However, whether its hypotensive effect will reduce long-term mortality remains unclear. Our study reported an exploratory follow-up of mortality outcomes from previous randomized controlled trial to assess the long-term effect of low-sodium salt on total and cardiovascular disease (CVD) mortality.

**Methods:** Participants who completed a previous 3-year double-blind randomized controlled trial were followed up from 2009 to 2019 to collect mortality data. Multivariable Cox regression models were used to evaluate the association between low-sodium salt intervention and all-cause and CVD mortality.

**Results:** Four hundred and forty participants completed the intervention trial, of which 428 participants had death outcome data recorded after 10 years follow-up: 209 in a salt substitute group and 219 in a normal salt group. Fifty participants died during follow-up, 25 died due to CVD. No significant differences in relative risks were found for all-cause mortality [HR = 0.81, 95% confidence interval (CI): 0.46–1.42] and CVD mortality (HR = 0.58, 95% CI: 0.26–1.32) in unadjusted analyses. After adjusted with age and alcohol drinking status, there were significant reductions for stroke mortality among all participants (HR = 0.26, 95% CI: 0.08–0.84) and for CVD mortality (HR = 0.38, 95% CI: 0.16–0.92) and stroke mortality (HR = 0.25, 95% CI: 0.08–0.82) among hypertensive participants.

**Conclusions:** Compared to normal salt, salt substitute might reduce the risk of CVD death, especially stroke among hypertensive patients. Our exploratory follow-up results provide potential evidence that low-sodium salt may be an accessible and effective strategy for prevention of CVD events, but definitive randomized controlled trials are warranted.

## Introduction

According to the Global Burden of Disease (GBD) report, cardiovascular disease (CVD) is the leading cause of death globally, accounting for 17.8 million deaths in 2017 (1). Similarly, in China, the incidence and consequent mortality of CVD has been increasing (2). It is widely recognized that excessive sodium consumption increases blood volume and the resistance of peripheral vessels, resulting in raised blood pressure and CVD (3, 4). Salt is the main dietary source of sodium. Salt reduction is seen as the most cost-effective public health strategy for preventing hypertension and CVD in developed and developing countries (5, 6). In China, due in part to traditional dietary habits, salt consumption has been shown to be the highest in the world, with adults consuming on average over 10 grams of salt daily (12.9 g/d in 1992, 12 g/d in 2002, and 10.5 g/d in 2015) (7): over twice the WHO (World Health Organization) recommended limit (5 g/d) (8). It is therefore imperative to explore suitable strategies for salt reduction in China, without undue changes to dietary habits and culture.

Salt substitutes, as an existing salt reduction strategy available in industrialized China, are formulations where a proportion of the sodium is replaced with potassium or other element. Compared with normal salt with 100% sodium chloride, salt substitutes seek to decrease sodium intake without reducing the perceived total salt consumption, and thus avoid the inherently poor compliance typical of long-term behavioral intervention in salt restriction (9, 10). In our study, the salt substitute used comprised 65% sodium chloride, 25% potassium chloride, and 10% magnesium sulfate. Since 1986, 20 articles have reported the effects of salt alternatives, with results focusing primarily on blood pressure and the incidence of hypertension (11, 12). Data on the population effect of sodium consumption on CVD or death is limited.

Our previous randomized, double-blind, controlled study provided evidence that an appropriate salt substitute could lower blood pressure during a 3 year intervention (13, 14). Whether this hypotensive effects might prove durable and decrease long-term CVD mortality remains unclear. Here, we report a 10-year post-intervention follow-up study to explore the long term effects of salt substitute and its effect on total and CVD mortality.

## Methods

### Study Design and Participants

Our study was an exploratory follow-up study with participants who completed the previous double-blind, randomized controlled trial. The previous trial explored the hypotensive effect of salt substitute. A detailed description of the previous trial has been reported previously (13, 14). Briefly, 200 families (462 participants) were randomized to an intervention (salt substitute) or control (normal salt) group in 2006. During intervention, participants were followed-up every 3 months to measure their systolic blood pressure (SBP) and diastolic blood pressure (DBP). Four hundred and forty participants completed the intervention in 2009. After completing the trial, participants were told the final results and both groups were recommended to reduce salt consumption to <6 g daily.

Subsequently, we have undertaken a 10-year observational study among the participants who completed the trial to examine the long-term effects of salt substitute on all-cause and CVD mortality. All surviving participants or relatives who provided information regarding deceased participants gave written informed consent. The institutional review board at the China Medical University approved the study. The study protocol conformed to the ethical guidelines of the 1975 Declaration of Helsinki, as reflected in an *a priori* approval by the institution's human research committee.

### Follow-Up and Assessment of Outcome

During the post-intervention follow-up, village health workers reviewed participants by telephone semi-annually. Multiple sources were analyzed to determine the time and cause of death, including medical records, death certificates and symptoms, as reported by a spouse, sibling, or child, were also cross-checked with the death registration system, where possible. Two physicians blinded to treatment, assessed the underlying cause of death, as obtained from death certificates, and assigned a code according to the International Classification of Disease, Tenth Revision (ICD-10). Causes of death were divided into two broad categories: CVD death (heart disease: I05-I09, I11, I20-I27, I30-I52; stroke: I60-I69) and non-CVD death (all other causes). The primary study endpoints were all-cause mortality and CVD mortality. The follow-up period started at the end of the intervention (April, 2009) lasting till death, loss to follow-up, or 30 April 2019, whichever occurred first.

### Statistical Analysis

Data were presented as mean ± standard deviation (SD) for continuous variables and as number (*n*) and percentage (%) for categorical variables. *T*-test and Chi-square test were used to compare differences in baseline characteristics. Cause-specific mortality for all-cause, CVD, and non-CVD deaths were calculated as incidence density and cumulative incidence. The efficacy of salt substitution on mortality was evaluated using absolute risk reduction (ARR), relative risk reduction (RRR), and number-needed-to-treat (NNT). Multivariate Cox proportional hazards models were used to obtain hazard ratios (HRs) and 95% confidence intervals (95% CIs) for mortality, adjusted for baseline age and alcohol consumption. All analyses were undertaken using SPSS statistical software (IBM SPSS Statistics for Windows, Version 23.0. Armonk, NY, USA). A 2-sided *P* < 0.05 was considered statistically significant.

## Results

### Study Population and Participants Characteristics

**Figure 1:**
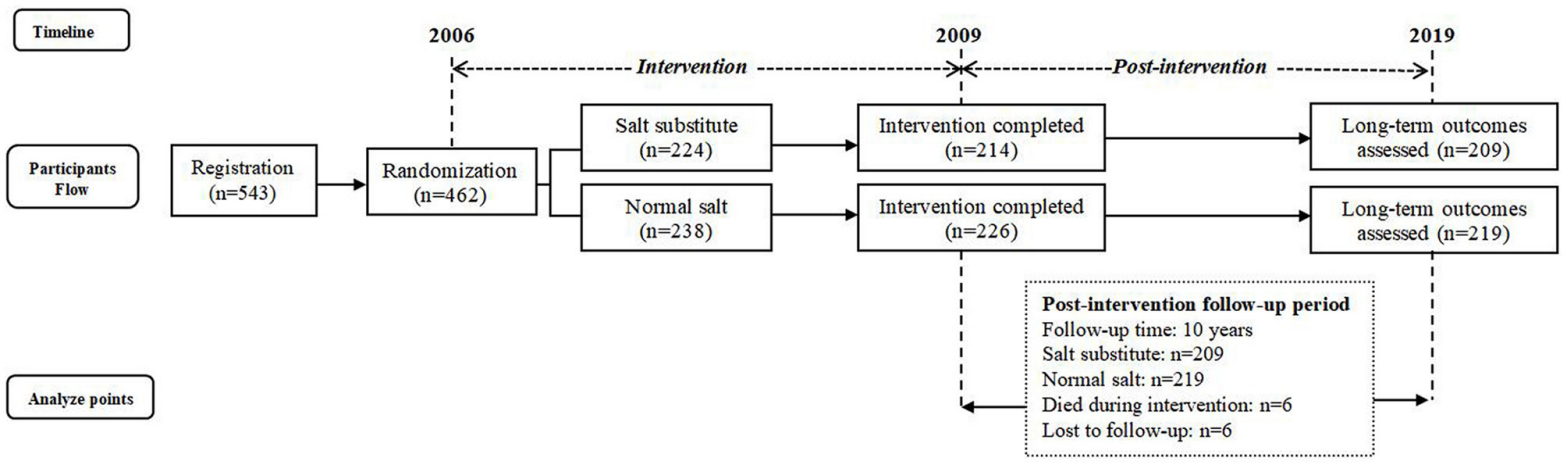
Flow diagram of study design and participant follow-up.

Of the 462 participants in the 2006 study, 224 were assigned to the salt substitute group and 238 to the normal salt group, with 440 participants completing the 3-year intervention. The principal reasons for participants lost to follow-up were those who moved beyond observation and others reluctant to follow the prescribed schedule. As 6 participants died during intervention and 6 participants could not be traced, valid follow-up information relating to death outcomes was obtained from 428 participants only, 209 in the salt substitute group and 219 in normal salt group (Figure 1). Characteristics of participants at baseline (2006) and the end of the intervention (2009) are shown in Table 1. At baseline, no differences were seen in terms of age, gender, BMI, smoking, history of hypertension, history of CVD, medication use, SBP, DBP, or pulse between salt substitute and control groups, except for alcohol consumption (*P* = 0.007). At the end of intervention period, differences were shown in medications use (*P* = 0.001) and SBP level (*P* = 0.049), and the change in SBP and DBP during the intervention period (2006–2009) differ significantly (*P* < 0.001) by groups.

**Table 1:** Characteristics of study participants by group at baseline (2006) and end of 3-year intervention (2009).

	**Salt substitute group**	**Normal salt group**	***P*-value**
**Baseline (2006)**			
Age (years)	45.4 ± 13.4	46.8 ± 13.2	0.283
Gender (male/female)	102/107	108/111	0.916
BMI (kg/m^2^)	26.0 ± 3.9	26.7 ± 4.28	0.062
Cigarette smoking [*n* (%)]	92 (44.0%)	84 (38.4%)	0.234
Alcohol drinking [*n* (%)]	90 (43.1%)	67 (30.6%)	0.007
Hypertension [*n* (%)]	160 (76.6%)	162 (74.0%)	0.536
CVD [*n* (%)]	37 (17.7%)	26 (11.9%)	0.089
Medications [*n* (%)]	87 (46.8%)	89 (46.1%)	0.897
SBP	154.6 ± 28.2	149.7 ± 23.5	0.053
DBP	92.0 ± 14.5	89.6 ± 13.8	0.079
Pulse (bpm)	79.3 ± 11.0	81.2 ± 12.2	0.083
**After intervention (2009)**			
BMI (kg/m^2^)	27.1 ± 3.7	26.52 ± 4.2	0.253
Cigarette smoking [*n* (%)]	64 (36.0%)	55 (29.6%)	0.194
Alcohol drinking [*n* (%)]	51 (29.1%)	47 (25.7%)	0.463
Hypertension [*n* (%)]	148 (82.7%)	159 (85.9%)	0.392
CVD [*n* (%)]	39 (21.8%)	33 (17.8%)	0.344
Medications [*n* (%)]	48 (26.8%)	81 (41.5%)	0.001
SBP	143.5 ± 21.4	148.4 ± 25.3	0.049
DBP	89.0 ± 12.8	91.2 ± 14.0	0.115
Pulse (bpm)	77.1 ± 10.7	77.0 ± 12.0	0.933
**Change for blood pressure from 2006 to 2009 (mmHg)**			
SBP	−14.3 ± 21.1	−5.4 ± 22.7	<0.001
DBP	−4.7 ± 12.0	−0.7 ± 13.4	<0.001

*CVD, Cardiovascular disease; BMI, Body mass index; SBP, Systolic blood pressure; DBP, Diastolic blood pressure*.

### Cause-Specific Mortality in Salt Substitute Group and Normal Salt Group

During the follow-up period, 50 deaths (22 in the salt substitute group, 28 in the salt group) were recorded, as shown in Table 2. Approximately 50% of deaths were due to CVD (heart disease and stroke) and 28% from cancer. A smaller proportion of participants died of all-cause mortality and CVD in the salt substitute group than in the control group, despite the difference not being statistically significant (all-cause: χ^2^ = 0.53, *P* = 0.467, CVD: χ^2^ = 1.75, *P* = 0.186). A marginally significant difference in stroke mortality was seen in participants taking salt substitute vs. normal salt (χ^2^ = 3.78, *P* = 0.052). A substantial risk reduction for stroke mortality was observed in the salt substitute group, as estimated by ARR and RRR. NNT for salt substitute intervention was 29. The non-CVD incidence of death per 1,000 person-years was 6.55 in the experimental group and 5.85 in the control group.

**Table 2:** Cause-specific mortality in groups and efficacy of salt substitute on mortality.

**Cause of death**	**Salt substitute group**			**Normal salt group**			**ARR^a^ (%)**	**RRR^b^ (%)**	**NNT^c^**
	**Death**	**Cumulative incidence (%; 95% CI)**	**Incidence density [deaths per 1,000 person-years (95% CI)]**	**Death**	**Cumulative incidence (%; 95% CI)**	**Incidence density [deaths per 1,000 person-years (95%CI)]**			
**All-cause**	22	10.53 (6.33 to 14.72)	11.08 (6.47 to 15.69)	28	12.79 (8.33 to 17.24)	13.66 (8.63 to 18.69)	−2.26	17.67	45
CVD	9	4.31 (1.53 to 7.08)	4.53 (1.58 to 7.49)	16	7.31 (3.83 to 10.78)	7.81 (3.99 to 11.62)	−3.00	41.04	34
Heart disease	5	2.39 (0.30 to 4.48)	2.52 (0.31 to 4.72)	4	1.83 (0.01 to 3.61)	1.95 (0.01 to 3.86)	0.56	30.60	179
Stroke	4	1.91 (0.04 to 3.79)	2.01 (0.04 to 3.99)	12	5.48 (2.44 to 8.52)	5.85 (2.55 to 9.16)	−3.57	65.15	29
non-CVD	13	6.22 (2.92 to 9.52)	6.55 (3.00 to 10.10)	12	5.48 (2.44 to 8.52)	5.85 (2.55 to 9.16)	0.74	13.50	136
Cancer	7	3.35 (0.89 to 5.81)	3.53 (0.92 to 6.13)	7	3.20 (0.85 to 5.54)	3.42 (0.89 to 5.94)	0.15	4.69	667
Diabetes mellitus	2	0.96 (−0.01 to 2.29)	1.01 (−0.01 to 2.40)	2	0.91 (−0.04 to 2.18)	0.98 (−0.38 to 2.33)	0.05	5.50	2,000
Accident	2	0.96 (−0.01 to 2.29)	1.01 (−0.01 to 2.40)	2	0.91 (−0.04 to 2.18)	0.98 (−0.38 to 2.33)	0.05	5.50	2,000
Others	2	0.96 (−0.01 to 2.29)	1.01 (−0.01 to 2.40)	1	0.46 (−0.44 to 1.36)	0.49 (−0.47 to 1.44)	0.50	108.70	200

*CVD, Cardiovascular disease; ARR, Absolute risk reduction; RRR, Relative risk reduction; NNT, Number needed to treat*.

a *Calculate as cumulative incidence of death in salt substitute group-cumulative incidence of death in normal salt group*.

b *Calculate as |ARR|÷cumulative incidence of death in normal salt group*.

c *Calculate as 1÷|ARR|*.

### Long-Term Effect of Salt Substitute Intervention Among All and Hypertensive Population

**Figure 2:**
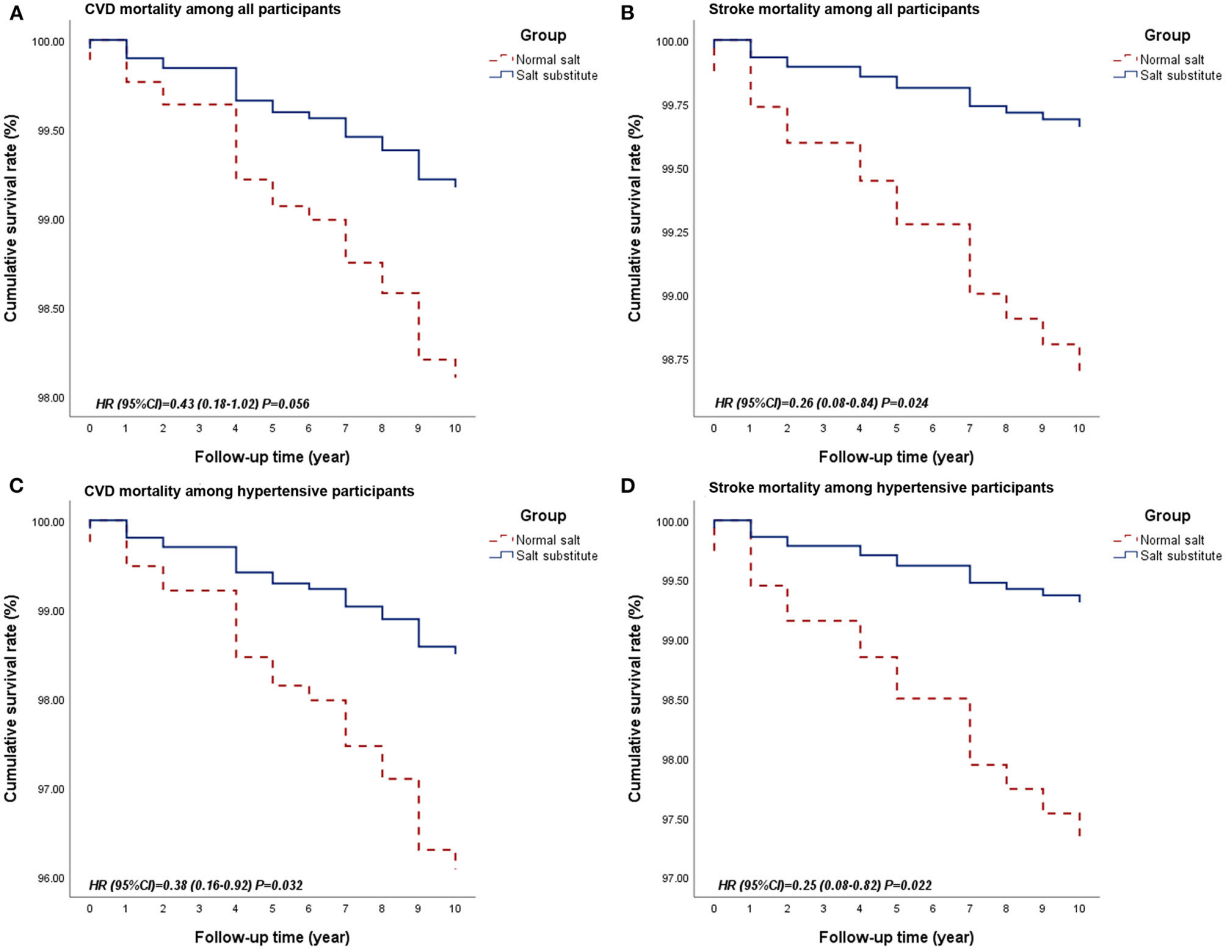
Survival curves of CVD and stroke mortality between groups among all participants or hypertensive participants. (**A**. Survival curve for CVD mortality among all participants; **B**. Survival curve for stroke mortality among all participants; **C**. Survival curve for CVD mortality among hypertensive participants; **D**. Survival curve for stroke mortality among hypertensive participants.) CVD, Cardiovascular disease.

Participants in the salt substitute group had improved survival for all-cause mortality and CVD-related mortality than controls. This protective effect was more obvious in hypertensive patients (Table 3 and Figure 2). In the multivariable regression adjusted for baseline age and alcohol consumption, salt substitute had an associated 57% (HR = 0.43, 95% CI = 0.18–1.02, *P* = 0.056; Figure 2A) and 62% (HR = 0.38, 95% CI = 0.16–0.92, *P* = 0.032; Figure 2C) lower risk of CVD death when compared to normal salt among all and hypertensive participants. Mortality risks from heart disease and stroke were lowered by 3 and 74% among all participants, 23 and 75% among hypertensive participants, while a statistically significant reduction was only found for stroke death (Figures 2B,D).

**Table 3:** Effect of salt substitute intervention on all-cause mortality and cardiovascular disease mortality among all and hypertensive participants.

**Cause of death**	**HR_**rude**_**	***P***	**HR_**adjust1**_**	***P***	**HR_**adjust2**_**	***P***
**All participants**						
All-cause	0.81 (0.46–1.42)	0.463	0.80 (0.46–1.40)	0.434	0.75 (0.42–1.32)	0.311
CVD	0.58 (0.26–1.32)	0.194	0.48 (0.21–1.13)	0.094	0.43 (0.18–1.02)	0.056
Heart disease	1.28 (0.35–4.79)	0.707	1.05 (0.27–4.12)	0.945	0.97 (0.24–3.88)	0.961
Stroke	0.35 (0.11–1.07)	0.066	0.29 (0.09–0.94)	0.039	0.26 (0.08–0.84)	0.024
**Hypertensive participants**						
All-cause	0.73 (0.41–1.30)	0.284	0.71 (0.40–1.28)	0.252	0.68 (0.38–1.22)	0.195
CVD	0.49 (0.21–1.15)	0.103	0.41 (0.17–0.99)	0.049	0.38 (0.16–0.92)	0.032
Heart disease	0.98 (0.25–3.92)	0.976	0.79 (0.19–3.37)	0.750	0.77 (0.18–3.34)	0.726
Stroke	0.33 (0.11–1.02)	0.055	0.28 (0.09–0.91)	0.034	0.25 (0.08–0.82)	0.022

*CVD, Cardiovascular disease; adjust1, Adjusted with baseline age; adjust2, Adjusted with baseline age and alcohol drinking status*.

### Measurements of Urinary Na^+^, K^+^, and Na^+^/K^+^ Ratios After Intervention

**Figure 3:**
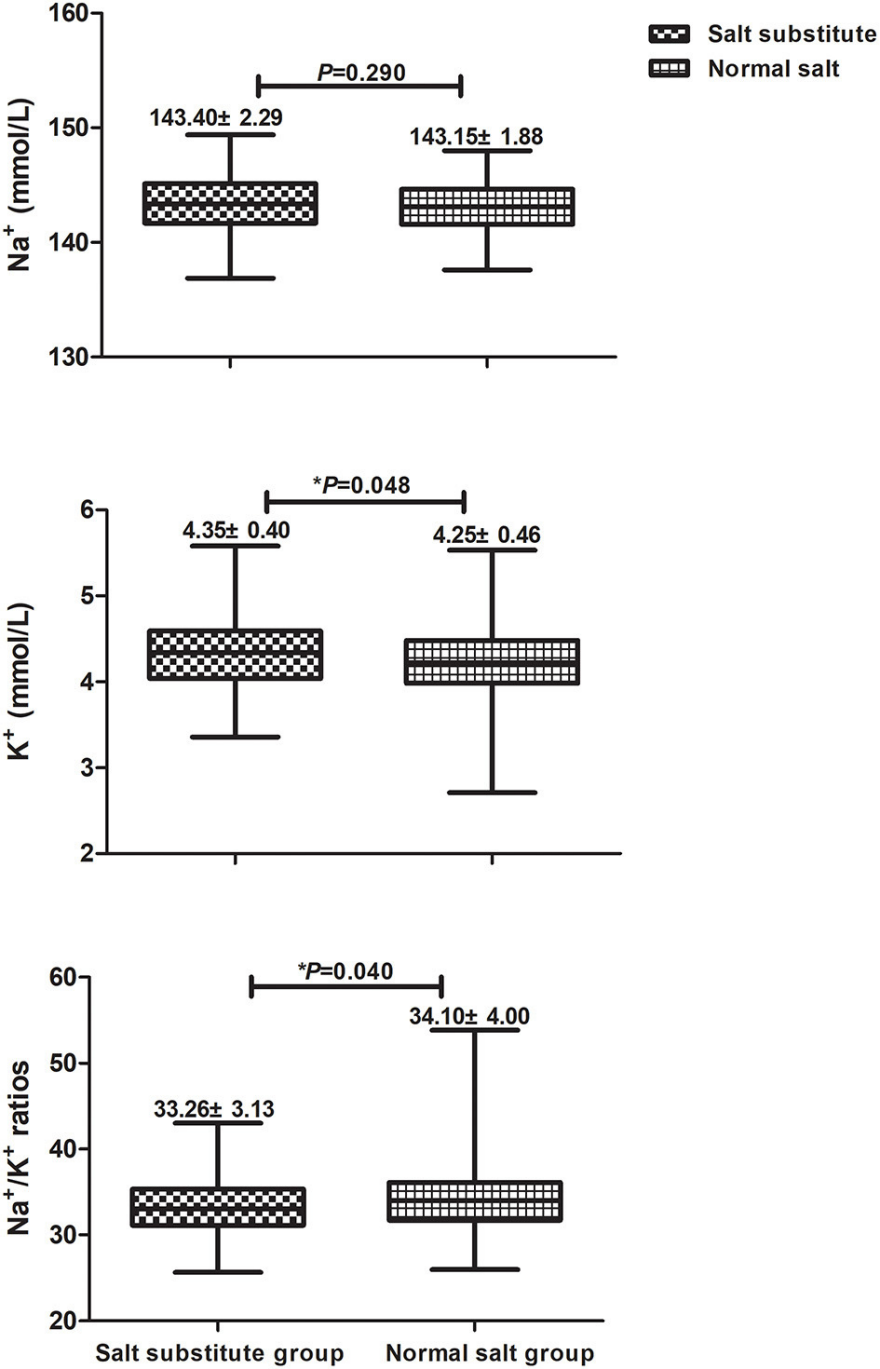
Comparisons for urinary Na^+^, K^+^, and Na^+^/K^+^ ratios between salt substitute group and normal salt group.

To evaluate treatment safety, spot urinary samples were collected and the concentration of Na^+^ and K^+^ at the end of intervention was measured. Urine data were available for 311 participants (161 salt substitute group, 150 normal salt group), as shown in Figure 3. The urinary K^+^ excretion and Na^+^/K^+^ ratios were significantly different between groups after 3-year intervention, but no difference was identified for urinary excretion of Na^+^.

## Discussion

The results of our previous RCT indicated salt substitute offered significant benefits for lowering SBP and DBP, with potential effects on hypertension (13, 14). However, the benefit of such intervention on cardiovascular disease and mortality was undetermined, necessitating a long-term follow-up study to evaluate the persistent effect of salt substitute. The results of our exploratory analysis following previous trial observed participants who received salt substitute had reduced total and CVD mortality, with larger effects observed in participants with baseline hypertension. Compared with the normal salt group, a statistically significant reduction (74%) in overall stroke mortality was observed, with reduced CVD (62%) and stroke mortality (75%) seen in hypertensive participants. This suggests that replacing normal salt with low-sodium salt lowers CVD and stroke mortality, particularly in individuals with hypertension.

Substantial evidence supports salt restriction as an effective non-pharmacological intervention at the population level for blood pressure management and improved long-term cardiovascular outcomes (11, 15–17). However, experience with behavior changing interventions shows even a moderate salt restriction for more than 6 months was hard to implement (9). Here, we used a commercially available salt substitute (18), which proved a pragmatic intervention strategy to reduce sodium intake (19). Hitherto, most salt substitution trials have evaluated blood pressure or hypertension as the primary outcome, with data concerning incidence, occurrence, and mortality of blood pressure-related cardiovascular events going unrecorded. Our study has reported the long-term effect of salt substitute on total and CVD mortality, indicating our study represents a significant advance.

Despite the long history of salt reduction research, associations between salt reduction and health outcomes remain at issue. In most studies, sodium intake was estimated form urinary sodium excretion. Follow-up of the famous TOHP (trials of hypertension prevention) report sodium reduction may reduce long-term CVD events (10–15 years after intervention) and all-cause mortality (23–26 years after intervention), suggesting a direct linear relationship between habitual sodium intake and total deaths (20–22). Meta-analysis and some observational studies reported increased CVD events at very low sodium intake, indicating a “J” or “U” shaped link between sodium intake and health outcomes (23–27). Results suggested an increasing CVD and mortality risk occurred at sodium intakes <3 g/day and >6 g/day (23), challenging the WHO's recommendation (<2 g/day of sodium, equivalent to 5 g salt/day), and querying the population-wide salt reduction policy to reduce blood pressure and CVD (8). However, methodological issues were readily apparent, mostly focusing on the use of suboptimal measurements (spot urine) to assess sodium consumption, as well as potential reverse causality in the studies, and many confounding factors (28, 29).

Stroke is strongly associated with blood pressure-lowering intervention (30), with salt reduction potentially reducing blood pressure, yet there is limited data from properly conducted randomized trials evaluating the beneficial effects of salt substitution on stroke. Pan et al.'s research reported improved outcomes for stroke recovery after 6 months' intervention with salt substitute (31). However, the results were questioned due to weaknesses in trial methodology (32). Here, we found a considerable association between low-sodium salt and reduced stroke risk in our long-term cohort study. However, given blood pressure control level has a great impact on the stroke incidence and mortality, and we did not monitor the blood pressure level during follow-up period. Therefore, the statistical association should be interpreted cautiously and still need a finding deserving the further research. The ongoing Salt Substitute and Stroke Study (SSaSS) enrolled 20,996 patients at elevated risk of stroke across 600 villages in rural China. This is designed to assess effects of salt substitution on fatal and non-fatal stroke and other CVD events. The trial will complete shortly and should provide further information about the efficacy and safety of salt substitution, giving anticipated support to global sodium reduction strategies (33).

There is also evidence that a high-salt diet might be particularly harmful in hypertensive patients (34). The DASH (Dietary Approaches to Stop Hypertension) study indicated that salt reduction was beneficial in both hypertensive and non-hypertensive individuals, with the greatest blood pressure decrease found in hypertensive patients (35). An updated Cochrane review also indicated that cardiovascular mortality were reduced by decreasing salt intake among hypertensives, though not in the general populations (36), which again was consistent with our results. Together, these results support strongly the greater effects of salt reduction in hypertensive patients.

To date, there are few reports of long-term trials evaluating the effect of sodium on clinical outcomes, primarily due to logistic and feasibility considerations. The longest intervention period of sodium reduction trials was 36 months (21), and the longest salt substitute intervention period was 44 months (37). Such trials were too short to observe mortality or cardiovascular disease events in the general population. In 2016, Cook et al. reported the over 20-year post-trial follow-up results to evaluate the relationship between sodium reduction intervention and total mortality, and a non-significant 15% lower risk was observed among participants in intervention group compared with controls (21). Furthermore, non-significant risk reduction also reported on CVD mortality and events (36), which was inconsistent with our findings. There are two possible explanations. One possible reason for this inconsistency might be the participants involved in TOHP trial were pre-hypertensive adults, but over 70% participants were hypertensive patients with increased likelihood of CVD events in present study. Another possible reason is the different intervention. Compared with salt restriction intervention in TOHP trial, salt substitute intervention could not only reduce sodium intake, but also increase potassium intake simultaneously. Meta-analysis and RCTs both found potassium-enriched salt substitutes, compared with normal salt, reduced blood pressure level and risk of death from cardiovascular disease (4, 11, 37). Thus, the protective effect of CVD mortality was more remarkable in our study.

The observed association between sodium reduction and total or CVD mortality can be explained by the “programming” effect. Geleijnse's demonstrated that infants given low sodium formula during their first 6 months had lower SBP than controls after 15 years follow-up, despite no corresponding difference in their urinary sodium excretion (38). This is also seen in adults. Among participants in the Baltimore TOHP I study, a trend for lower blood pressure and reduced incidence of hypertension was observed in the reduced sodium group compared to controls, despite no difference in urinary sodium excretion (35). One explanation of this might be that even though sodium reduction was short in duration, blood pressure regulation was reset and subsequent structural or functional damage to cardiac and vascular systems was delayed due to this initial sodium reduction. An alternative reason might be that participants in the intervention group could well adopt salt reduction and live a healthier lifestyle once they knew the results of the RCT indicating that salt substitution reduced blood pressure. Since our study did not record changes in dietary or behavior after intervention, further investigations are required to confirm or refute this hypothesis.

From the viewpoint of medical and financial demands on both government and patient, salt reduction is a cost-effective and promising strategy for reducing the burden of CVD. In China, driven by traditional cooking and eating habits, the long-term compliance with salt restriction is poor. Simply reducing the amount of salt consumed is not practical. The salt substitute we used comprised 65% NaCl, reducing the amount of sodium consumed by 35%, when compared to regular salt [100% NaCl]. Likewise, we found no statistically significant reduction in urinary Na^+^ in our study. Possible explanations include: (1) our previous study was undertaken in the general population and lasted 3 years, so it was not possible to restrict food intake fully. Thus, concentrations of urinary Na^+^ might be affected by other food contained large quantities of sodium, including *inter alia* monosodium glutamate, flour strings, and fermented bean curd. (2) The family baseline salt intake survey showed average salt consumption per participant to be 12.64 g/d (salt substitute group) and 9.36 g/d (normal salt group), indicating higher total salt consumption in the intervention group, possibly explaining why urinary Na^+^ was slightly higher in this group. Moreover, our findings identified significant differences in K^+^ excretion and Na^+^/K^+^ ratios, in line with expectations. Thus, substituting normal salt with potassium-enriched and low-sodium salt may prove effective in promoting healthier lifestyle among people without severely-impaired kidney function. Considering the results of urinary Na^+^ and K^+^, we make the assumption that the protective effect may primarily be due to the increase in potassium intake, because the sodium reduction achieved was moderate. Additional studies are encouraged to clarify whether the effect comes from lower sodium intake or from higher potassium intake.

Because the exploratory nature of present analysis, our previous trial was not originally designed to evaluate the impact of salt substitute intervention on survival outcome, the design of this follow-up study had flaws and statistical power was limited. First, our study did not evaluate sodium intake during the follow-up period, preventing identification of participants who continued to use salt substitutes or tried to restrict salt intake following our post-intervention recommendations. This means any observed benefits of low-sodium salt might result from other lifestyle change during follow-up. People who try one type of lifestyle intervention (sodium reduction) may be more motivated to try other health interventions, such as exercise, eating more fruit and vegetables, reducing smoking or alcohol consumption, etc. Unfortunately, such data could not be factored into our analysis. Secondly, because the information was collected by telephone interview, the blood pressure data, which have enormous implications for the progression of cardiovascular disease, were failed to collect in our follow-up study. Third, since over 70% of participants were hypertensive patients and the small number of deaths among those without hypertension, we could not evaluate properly if low-sodium salt reduced total and CVD mortality in the non-hypertensive population. Additionally, considering the low mortality level, we could only adjust baseline age and alcohol drinking status, which may affect the accuracy of our results.

## Conclusions

Our results provide evidence that replacing normal salt with a low-sodium salt substitute could be a tractable and effective strategy to prevent CVD events. Salt substitute, which typically lowers blood pressure and prevents hypertension, may thus reduce CVD mortality, especially in hypertensive participants. Well-designed multi-center RCTs accessing large, properly-stratified patient populations will be required to elucidate properly and completely the effects of salt substitution on cardiovascular outcomes in the population.

## Data Availability Statement

The raw data supporting the conclusions of this article will be made available by the authors, without undue reservation.

## Ethics Statement

The studies involving human participants were reviewed and approved by the institutional review board at the China Medical University. The patients/participants provided their written informed consent to participate in this study.

## Author Contributions

BZ contributed to the conception and design of the work. HS, BM, and XW contributed to the acquisition, analysis, or interpretation of data for the work. HS drafted the manuscript. HW revised the manuscript. All authors gave final approval and agree to be accountable for all aspects of work ensuring integrity and accuracy.

## Conflict of Interest

The authors declare that the research was conducted in the absence of any commercial or financial relationships that could be construed as a potential conflict of interest.
